# Remnant cholesterol: an independent, dose-dependent risk factor for hyperuricemia in a normolipidemic chinese population

**DOI:** 10.3389/fendo.2025.1718817

**Published:** 2026-01-12

**Authors:** Huali Xiong, Fengxun Ma

**Affiliations:** 1 Department of Chronic Noncommunicable Disease Prevention and Control, Center for Disease Control and Prevention of Rongchang District, Chongqing, China; 2 Department of Public Health, The People’s Hospital of Rongchang District, Chongqing, China

**Keywords:** remnant cholesterol, hyperuricemia, uric acid, normal lipid profiles, CMEC

## Abstract

**Backgroud:**

The association between remnant cholesterol (RC) and hyperuricemia (HUA) remains unclear in the general population with entirely normal lipid profiles and no prior lipid-lowering therapy. This study aimed to investigate the association between RC and HUA in a normolipidemic Chinese population.

**Method:**

We recruited 2,171 participants aged 30–79 years from Rongchang, Chongqing Municipality, southwest China, as part of the China Multi-Ethnic Cohort Study. Logistic regression, restricted cubic spline (RCS), and mediation analyses were applied to evaluate the association between RC and HUA. Furthermore, subgroup and sensitivity analyses were conducted to assess the consistency of the findings.

**Results:**

A total of 2,171 participants were enrolled, with a mean (SD) age of 50.43 (12.21) years. The overall prevalence of HUA was 9.07%, and the prevalence across RC quartiles (Q1: 0.14–0.43, Q2: 0.44–0.60, Q3: 0.61–0.89, and Q4: ≥0.90 mmol/L) were increased stepwise: 4.45%, 8.32%, 12.88%, and 19.08%, respectively(*P*_for trend_ < 0.001). Logistic regression revealed that the ORs were 1.753 (95%*CI*: 1.093–2.809), 2.900 (95%*CI*: 1.845–4.558), and 4.268 (95%*CI*: 2.373–7.674) in Q2, Q3, Q4, respectively, compared to Q1 after adjusting for confounding factors. RCS revealed that RC was positively associated with HUA by a linear model (*P*_for overall_ < 0.001, *P*_for nonlinear_>0.05), with no evidence of a threshold. These findings remained robust in sensitivity analyses that excluded participants with hypertension, diabetes, overweight/obesity or central obesity. Subgroup analyses revealed consistent RC-HUA associations across strata of age, sex, smoking status, drinking status, hypertension status, diabetes status, overweight/obesity status, central obesity status, DASH score. Mediation analyses revealed a potential mediation effect between RC and uric acid(UA), indicating that IR may mediated 39.91% of the total association between RC and UA.

**Conclusion:**

Remnant cholesterol emerges as a fully independent, dose-dependent, and readily modifiable determinant of incident hyperuricemia in Chinese adults, which can serve as an ideal candidate for inclusion in routine metabolic panels and presenting as a hypothesis for future longitudinal research.

## Introduction

Hyperuricemia(HUA), resulting from disordered purine metabolism, is the biochemical hallmark and direct causative factor of gout. The pathogenesis of HUA primarily stems from dynamic balance of uric acid(UA) homeostasis, which can be attributed to two main pathways (1, 2): first, endogenous purine metabolism dysregulation leading to excessive uric acid production; and second, impaired renal uric acid excretion resulting in reduced uric acid clearance. Moreover, it serves as an independent risk factor for hypertension, diabetes, chronic kidney disease, cardiovascular disease, and stroke (3). In China, the prevalence of HUA has been increasing annually and is shifting toward younger populations, currently ranking as the second most common metabolic disorder after diabetes (4).

The risk factors for HUA have been extensively investigated. For instance, hypertension, obesity, and inflammatory responses have been identified as key predisposing factors (5–7). Dietary westernisation and increasing obesity have elevated serum uric acid(SUA) levels even in “apparently healthy” adults with guideline-concordant lipid profiles, among whom the prevalence of HUA remains 10–20% (8). Historically, studies on the association between blood lipids and hyperuricemia have primarily focused on traditional lipid parameters, particularly triglycerides (TG) and high-density lipoprotein cholesterol(HDL-C) (9–11). A consistent and significant association has been widely observed between these two lipid markers and hyperuricemia. Therefore, identifying modifiable lipid-related indicators for the early detection of potential hyperuricemia in metabolically normal individuals has become a key prevention priority.

Remnant cholesterol(RC) is comprised of the cholesterol liberated from triglyceride-rich lipoproteins(TGRLs) that remains following intravascular lipolysis (12), which can directly penetrate the vascular endothelium, accumulate within the vascular wall, and trigger inflammatory responses (13). It can further disrupt purine metabolism and inhibit renal urate excretion, thereby promoting hyperuricemia. Recent cardiovascular outcome data indicate that the risks of coronary heart disease and atherosclerosis persist even in patients with optimal LDL-C levels. RC has been increasingly implicated in this residual risk, becoming a focus of research aimed at explaining its underlying cause (14, 15). As an emerging, non-traditional lipid parameter, elevated RC exhibits a strong, independent, and positive association with HUA (16, 17). Elevated RC is significantly associated with HUA in patients with type 2 diabetes mellitus, and RC demonstrates superior predictive value and outperforms traditional lipid metrics (18). While the positive correlation between RC and HUA has been established by previous studies, the existing evidence is primarily derived from studies in populations with hypertriglyceridemia or metabolic syndrome. Consequently, these studies cannot answer a more public health–relevant question: whether RC independently promotes hyperuricemia in the general population with entirely normal lipid profiles and no prior lipid-lowering therapy remains untested.

However, direct epidemiological evidence validating the association between RC and HUA remains scarce at the population epidemiological level. The few existing observational studies are often confounded by use of lipid-lowering agents, urate-lowering medications, as well as complex dyslipidemia conditions, making it difficult to establish an independent association between RC and HUA. Additionally, traditional lipid metrics (LDL-C, TG, HDL-C) lose predictive value for hyperuricemia when their levels fall within the reference range. RC, a non-fasting marker of residual atherogenic burden, may provide information beyond these conventional measures. Furthermore, prior studies have implicated a positive feedback loop between RC and HUA, with insulin resistance (IR) serving as a key mediator. RC directly fosters IR and, in concert with IR, synergistically accelerates HUA progression (16, 19).

We hypothesize that RC relates to HUA independently of dyslipidemia or lipid drugs and could serve as an early biomarker for occult hyperuricemia risk stratification in the general population. This study aims to: (1) investigate the association between RC levels and HUA risk; (2) characterize the dose-response association between RC and HUA; (3) perform stratified analyses by sex, age, body mass index (BMI), and other factors to verify the robustness of these estimates; and (4) conduct mediation analyses to quantify the contribution of IR, with the goal of elucidating a “direct crosstalk” between RC and UA rather than a “secondary echo of metabolic disorders.” The findings will clarify lipid-centric pathways underlying urate dysregulation and offer a readily modifiable biomarker for the early identification of “metabolically silent” HUA risk.

## Method

### Study design

The study participants were recruited from the baseline cross-sectional survey of the China Multi-Ethnic Cohort(CMEC) study (20), a large community-based cohort established by Sichuan University in Southwestern China from September 2018 to January 2019. Specifically, in the Rongchang area, a three-stage stratified random sampling approach was implemented for recruitment and the detailed sampling methodology has been published elsewhere (21). This study was approved by the Ethics Committee of Sichuan University(No. K2016038), and written informed consents were obtained from all participants prior to their enrollment in the survey.

### Study population

**Figure 1:**
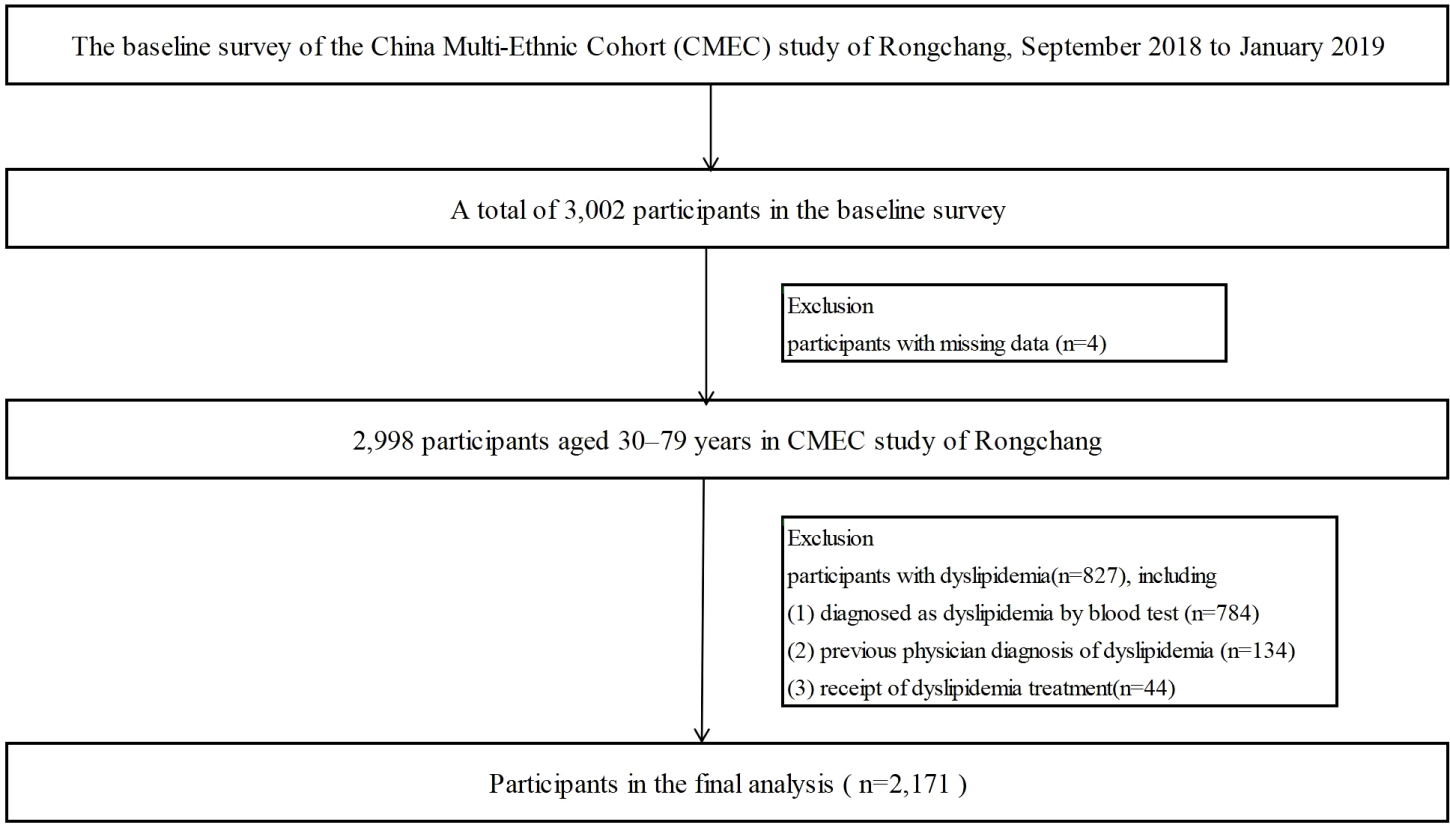
Flowchart on the sample selecting method at each step.

Participants in this study were enrolled according to the following inclusion criteria: (1) aged 30–79 years in 2018; (2) resided in Rongchang for over six months; (3) Han ethnicity; (4) voluntary participation, willingness to donate biological samples, and agreement to undergo follow-up assessments; and (5) no history of mental illness, cognitive impairment, or communication barriers. The exclusion criteria were: (1) a diagnosis of dyslipidemia; (2) current use of lipid-lowering medications; (3) current use of gout medications or diuretics that may affect uric acid levels; and (4) participants with incomplete data regarding demographics, questionnaires, physical examinations, or blood biochemical analyses were excluded. Consequently, the final analytical cohort consisted of 3,002 participants in the baseline survey. Among these, four participants were excluded due to missing data, 827 participants with dyslipidemia were excluded, and 2,171 participants were included in the final analysis (**Figure 1**).

### Data collection

#### Assessment of covariates

The survey was conducted through face-to-face interviews and comprised three main components: questionnaire administration, physical examinations, and laboratory tests. Well-trained interviewers collected data on demographic characteristics and lifestyle factors, including age, sex, marital status, educational level, occupation, total family income, smoking status, drinking status, physical activity (PA) levels, Dietary Approaches to Stop Hypertension (DASH) scores, and night sleep duration. Additionally, physical examination data, including systolic and diastolic blood pressure (SBP/DBP), height, body weight, and waist circumference (WC) were collected. Venous blood samples were obtained after an 8–hours fasting period (more than 8 hours after the last meal) to measure fasting blood glucose (FBG), total cholesterol (TC), triglycerides (TG), high-density lipoprotein cholesterol (HDL-C), low-density lipoprotein cholesterol (LDL-C), and uric acid (UA).

Age was categorized into three groups:30–44 years, 45–59 years and 60–79 years. Sex was dichotomized as males or females. Marital status was categorized into two groups: married/cohabiting, separated/divorced/widowed/unmarried. Educational level was grouped into three levels: primary school or below, junior middle school, and senior high school or above. Occupation was classified into five categories: farmers, government employees, sales staff, and others. Total family income was stratified into four brackets: <20,000 yuan, 20,000–59,999 yuan, 60,000–99,999 yuan, and ≥100,000 yuan. Smoking status was dichotomized as “No” or “Yes” based on lifetime cigarette consumption ≥100 cigarettes. Drinking status was also dichotomized as “No” or “Yes,” determined by whether participants had consumed alcohol in the 30 days prior to the survey.

PA levels were assessed using metabolic equivalent tasks (METs) (22), which accounted for PA engagement across four domains: work, transportation, household chores, and leisure activities. Total weekly PA volume was categorized based on MET-minutes per week (MET-min/w): <600 MET-min/w was defined as low, 600–3,000 MET-min/w as moderate, and >3,000 MET-min/w as vigorous (23).

Night sleep duration was categorized in accordance with guidelines from the sleep guidelines of national lung and blood institute, which specifies the recommended daily sleep duration for adults as 7–8 hours/day (24). Specifically, sleep duration was classified into three groups: <7 h/day (Insufficient), 7–8.9 h/day (Sufficient), and ≥9 h/day (Excessive).

A modified DASH scores were calculated following the method developed by Chiu (25), with minor adaptations based on data from the CEMC study (26). This modified score focused on 7 key food groups/components: whole grains, fresh fruits, fresh vegetables, legumes, dairy products, red meat, and sodium intake. Scores of 1–5 were assigned to each food group/component based on the quintiles of participants’ average weekly intake. Specifically, a score of 5 was assigned to the highest quintile of intake for beneficial food groups (e.g., whole grains, fresh fruits, fresh vegetables, legumes, and dairy products) and, conversely, to the lowest quintile of intake for unfavorable components (e.g., red meat and sodium). Each participant’s overall DASH score was subsequently calculated by summing the scores across all seven components (27). Using tertiles of the overall DASH score as the cutoff, participants were categorized into three groups: those with scores ≤20, 21–24 and ≥25.

Hypertension was defined as (1): an average SBP ≥140 mmHg or average DBP ≥90 mmHg (28)(based on three consecutive measurements taken at 5-minute intervals with the participant at rest); or (2) a previous physician diagnosis of hypertension; or (3) receipt of blood pressure-lowering treatment (e.g., medications, lifestyle modifications). Diabetes was defined as: (1) a fasting blood glucose (FBG) level ≥7.00 mmol/L; or (2) a previous physician diagnosis of diabetes; or (3) receipt of diabetes treatment (e.g., medications, lifestyle modifications) (29). Dyslipidemia was diagnosed if any of the following four lipid abnormalities were present: (1)TC≥6.2 mmol/L or/and TG≥2.3 mmol/L or/and LDL-C≥4.1 mmol/L or/and HDL-C<1.0 mmol/L (30); (2) a previous physician diagnosis of dyslipidemia; or (3) receipt of dyslipidemia treatment. Overweight and obesity were defined based on body mass index (BMI), which was calculated as body weight divided by the square of height (kg/m²). According to the Expert Consensus on Obesity Prevention and Treatment in China (31), participants with a BMI of 24.0–27.9 kg/m² were classified as overweight, and those with a BMI ≥28.0 kg/m² as obesity. Central obesity was defined based on waist circumference (WC): males with a WC ≥90 cm and females with a WC ≥85 cm were classified as having central obesity (32). Hyperuricemia was defined as a serum uric acid (UA) level ≥420 μmol/L (33).

#### Definition and calculation of remnant cholesterol

Remnant cholesterol (RC) refers to the cholesterol component of remnant lipoproteins, which is calculated using the formula: RC = TC − HDL-C − LDL-C (34). For reference, non-high-density lipoprotein cholesterol (Non-HDL-C), a distinct lipid parameter is calculated as: Non-HDL-C = TC − HDL-C (35). RC concentrations were stratified by quartiles (Q) as follows: Q1 (lowest), 0.14–0.43 mmol/L, Q2: 0.44–0.60 mmol/L, Q3: 0.61–0.89 mmol/L, Q4 (highest): ≥0.90 mmol/L.

#### Definition of insulin resistance

Insulin resistance was assessed using the Triglyceride-Glucose-Body Mass Index (TyG-BMI), a composite marker that integrates metabolic and anthropometric parameters to enhance the accuracy of insulin resistance evaluation (36). The calculation of TyG-BMI involves two sequential steps:First, compute the Triglyceride-Glucose (TyG) index: The TyG index is calculated using the natural logarithm of the product of triglyceride (TG) and fasting blood glucose (FBG) levels, divided by 2. The formula is: TyG index = ln[TG (mg/dL) × FBG (mg/dL)/2]. Second, calculate the TyG-BMI: TyG-BMI is derived by multiplying the TyG index by body mass index (BMI, kg/m²). The formula is: TyG-BMI = TyG index × BMI (kg/m²). This composite index is widely used in epidemiological studies to assess insulin resistance when direct measurements (e.g., hyperinsulinemic-euglycemic clamp) are unavailable, due to its simplicity and robust, validated association with key biomarkers of insulin resistance (37). IR was categorized into four groups based on quartiles. Q1:<18.14; Q2: 18.14—25.63; Q3: 25.64—35.81; Q4: ≥35.82.

#### Quality control

The Center for Disease Control and Prevention of Rongchang District constituted a dedicated investigation unit. All field staff completed systematic training and passed a certifying examination before deployment. standardized procedures for administering questionnaires via tablet devices, verifying and uploading questionnaire data, performing physical examinations, and collecting and transporting biological samples. Graduate students from Sichuan University performed dynamic on-site monitoring, randomly re-examining 1% of daily records to detect, report, and rectify discrepancies immediately. Physical examinations were conducted exclusively by licensed associate-chief physicians; blood collection, processing, and storage were executed by technicians holding associate-chief technologist rank or higher. All serum and urine analyses were undertaken by the third-party Chongqing Dian Medical Laboratory Center Co., Ltd.

### Statistical analyses

**Figure 2:**
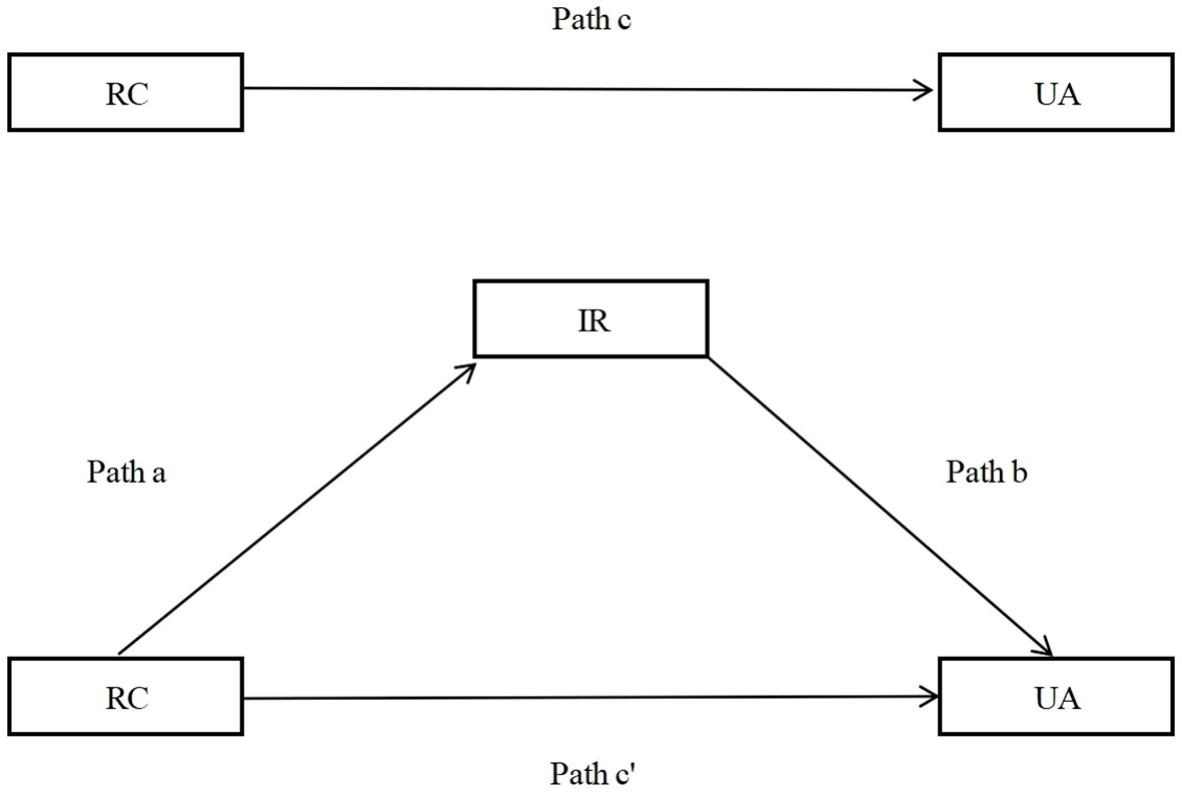
The detailed framework of mediation analysis of IR on the association between RC and UA. RC. remnant cholesterol. IR. insulin resistance; UA. uric acid.

Continuous variables with a normal distribution were expressed as mean ± standard deviation (SD), whereas those with a non-normal distribution were reported as median [interquartile range (IQR)]. Categorical variables were summarized as counts and percentages (%). The chi-square test was used to compare the prevalence of HUA and IR across participant characteristics. Logistic regression analyses were conducted to examine the association between RC (stratified by quartiles: Q1 as reference, Q2, Q3, Q4) and HUA, with odds ratios (ORs) and corresponding 95% confidence intervals (CIs) calculated. Four sequential models were constructed to adjust for potential confounding factors: Model 1: Unadjusted (no confounding factors included). Model 2: Adjusted for basic demographic factors: age, sex, marital status, educational level, occupation, and total household income. Model 3: Further adjusted for lifestyle factors: smoking status, drinking status, PA level, night sleep duration, and DASH score. Model 4: Additional adjustment for comorbidities and anthropometric indicators: hypertension, diabetes, overweight/obesity, and central obesity. Collinearity diagnostics were performed for all variables entered into the logistic regression model; multicollinearity was deemed absent if the variance inflation factor (VIF) was <5. To examine the association between RC and HUA, crude and multivariable-adjusted restricted cubic splines (RCS) were applied. Two key parameters were used to assess the performance of the RCS analysis, including *P*_for overall_ and *P*_for non-linear_. P _for overall_ was used to evaluate the significance of overall association between RC and HUA (combined linear and nonlinear components), with a *P*-value < 0.05 supported a significant association between RC and HUA and *P*_for non-linear_ was used to test the significance of the nonlinear component: a *P*-value ≥ 0.05 supported a predominantly linear relationship, whereas *P* < 0.05 justified retention of the nonlinear model. To evaluate the robustness of the associations, a sensitivity analyses was conducted by excluding participants with any of the following conditions: hypertension, diabetes, overweight/obesity, or central obesity. Logistic regression and RCS analyses were subsequently repeated after re-adjusting for the remaining confounding factors to reassess the association. Stratified logistic regression analyses and RCS were further performed to explore potential effect modifications of the RC–HUA association across subgroups, including age, sex, smoking status, drinking status, hypertension, diabetes, overweight/obesity, and central obesity. Furthermore, we employed mediation analysis to explore the potential mediation effect of IR in the association between RC and HUA by “mediation” package. In the mediation analysis, path c represents the total effect of RC on UA. Path a denotes the effect of RC on the mediator (IR), and path b denotes the effect of IR on UA. After controlling for IR, the effect of RC on UA is path c′. The indirect effect of RC on UA through IR is calculated as a × b. The proportion of mediation is computed as (indirect effect/total effect) × 100% (38). The detailed framework of mediation analysis is presented in **Figure 2**. All statistical analyses were performed using R software (version 4.3.1). A two-sided *P* value < 0.05 was considered statistically significant.

## Results

### Baseline characteristics of the study participants

A total of 2,171 participants were enrolled, with a mean (SD) age of 50.43 (12.21) years; 977 participants (45.92%) were males. Most participants were married or cohabiting (n=1,965, 90.52%), and 1,023 participants (47.12%) had completed high school or above education. Farmers was the primary occupation for 705 (32.47%) participants. The most frequently reported annual family income bracket was 20,000-59,999 yuan, representing 790(26.39%) of the participants (**Table 1**).

**Table 1:** Basic characteristics of the study participants by hyperuricemia [n (%)].

Variables	Total (n = 2,171)	Hyperuricemia		Statistic	*P*
		No (n = 1,974)	Yes (n = 197)		
age/year				4.152	0.125
30—44	740 (34.09)	679 (91.76)	61 (8.24)		
45—59	858 (39.52)	786 (91.61)	72 (8.39)		
60—79	573 (26.39)	509 (88.83)	64 (11.17)		
Sex				145.789	<0.001
Males	997 (45.92)	826 (82.85)	171 (17.15)		
Females	1,174 (54.08)	1,148 (97.79)	26 (2.21)		
Marriage status				1.432	0.232
Married/cohabiting	1,965 (90.51)	1,782 (90.69)	183 (9.31)		
Separated/divorced/widowed/unmarried	206 (9.49)	192 (93.20)	14 (6.80)		
Education level				1.956	0.376
Primary school or below	521 (24.00)	466 (89.44)	55 (10.56)		
Junior middle school	627 (28.88)	571 (91.07)	56 (8.93)		
High school or above	1,023 (47.12)	937 (91.59)	86 (8.41)		
Occupation				5.497	0.240
Farmers	705 (32.47)	650 (92.20)	55 (7.80)		
Government employees	233 (10.73)	205 (87.98)	28 (12.02)		
Workers	204 (9.40)	181 (88.73)	23 (11.27)		
Sales staff	371 (17.09)	341 (91.91)	30 (8.09)		
Others	658 (30.31)	597 (90.73)	61 (9.27)		
Total family income/yuan				1.472	0.689
<20000	671 (30.91)	613 (91.36)	58 (8.64)		
20,000—59,999	790 (36.39)	723 (91.52)	67 (8.48)		
60,000—99,999	372 (17.13)	334 (89.78)	38 (10.22)		
≥100,000	338 (15.57)	304 (89.94)	34 (10.06)		
Smoking status				29.788	<0.001
No	1,793 (82.59)	1,658 (92.47)	135 (7.53)		
Yes	378 (17.41)	316 (83.60)	62 (16.40)		
Drinking status				11.259	<0.001
No	1,085 (49.98)	1,009 (93.00)	76 (7.00)		
Yes	1,086 (50.02)	965 (88.86)	121 (11.14)		
PA level				0.094	0.954
Low	297 (13.71)	270 (90.91)	27 (9.09)		
Moderate	337 (15.55)	308 (91.39)	29 (8.61)		
Vigorous	1,533 (70.74)	1,393 (90.87)	140 (9.13)		
Night sleep duration				4.971	0.083
Insufficient	559 (25.75)	498 (89.09)	61 (10.91)		
Sufficient	1,392 (64.12)	1,280 (91.95)	112 (8.05)		
Excessive	220 (10.13)	196 (89.09)	24 (10.91)		
Dash score				1.350	0.509
≤20	725 (33.39)	652 (89.93)	73 (10.07)		
21—24	628 (28.93)	573 (91.24)	55 (8.76)		
≥25	818 (37.68)	749 (91.56)	69 (8.44)		
Hypertension				18.895	<0.001
No	1,398 (64.39)	1,299 (92.92)	99 (7.08)		
Yes	773 (35.61)	675 (87.32)	98 (12.68)		
Diabetes				2.696	0.101
No	2,008 (92.49)	1,820 (90.64)	188 (9.36)		
Yes	163 (7.51)	154 (94.48)	9 (5.52)		
Overweight/obesity				22.249	<0.001
No	1,053 (48.50)	989 (93.92)	64 (6.08)		
Yes	1,118 (51.50)	985 (88.10)	133 (11.90)		
Central obesity				22.976	<0.001
No	1,647 (75.86)	1,525 (92.59)	122 (7.41)		
Yes	524 (24.14)	449 (85.69)	75 (14.31)		
RC				49.091	<0.001
0.14—0.43	741 (34.13)	708 (95.55)	33 (4.45)		
0.44—0.60	649 (29.89)	595 (91.68)	54 (8.32)		
0.61—0.89	629 (28.97)	548 (87.12)	81 (12.88)		
≥0.90	152 (7.00)	123 (80.92)	29 (19.08)		

DASH. dietary approaches to stop hypertension; RC. remnant cholesterol; PA. physical activity; Statistic. analysed by chi square test.

### The HUA prevalence of the study participants

The overall prevalence of HUA was 9.07%. HUA frequency differed significantly by sex, smoking status, drinking status, hypertension, overweight/obesity, central obesity and RC levels (all *P* < 0.05). Across increasing RC quartiles (Q1: 0.14-0.43, Q2: 0.44-0.60, Q3: 0.61-0.89 and Q4: ≥0.90 mmol/L), the prevalence of HUA rose progressively: 4.45%, 8.32%, 12.88% and 19.08%, respectively (*P*_for trend_ < 0.001) (**Table 1**).

### The association between RC and HUA by logistic regression

In the unadjusted model, participants in the Q2(RC 0.44-0.60 mmol/L), Q3(0.61-0.89 mmol/L) and Q4(≥0.90 mmol/L) had progressively higher ORs of HUA, the ORs were 1.947 (95%*CI*:1.246-3.043), 3.171 (95%*CI*: 2.084-4.826) and 5.058 (95%*CI*: 2.965-8.631), respectively, compared with Q1(0.14-0.43 mmol/L) as reference(*OR* = 1) (**Supplementary Table 1**). After multivariable adjustment for age, sex, marriage status, education level, occupation, total family income, smoking status, drinking status, PA level, night sleep duration, Dash score, hypertension, diabetes, overweight/obesity, central obesity, these associations remained significant in Q2, Q3 and Q4, the ORs were 1.753 (95% *CI*: 1.093-2.809), 2.900 (95%*CI*: 1.845-4.558) and 4.268 (95%CI: 2.373-7.674), respectively (**Table 2**; details of the model 2 and model 3 were provided in **Supplementary Tables 2**–**4**). All variables entered into the logistic regression exhibited VIF <5, confirming the absence of multicollinearity.

**Table 2:** Association between remnant cholesterol and hyperuricemia by logistic regression, [OR(95%CI)].

Variables	Model 1	Model 2	Model 3	Model 4
RC				
0.14—0.43	1.000 (Reference)	1.000 (Reference)	1.000 (Reference)	1.000 (Reference)
0.44—0.60	1.947 (1.246-3.043)^*^	1.854 (1.170-2.937)^*^	1.860 (1.169-2.960)^*^	1.753 (1.093-2.809)^*^
0.61—0.89	3.171 (2.084-4.826)^**^	3.193 (2.063-4.943)^**^	3.273 (2.108-5.081)^**^	2.900 (1.845-4.558)^**^
≥0.90	5.058 (2.965-8.631)^**^	4.729 (2.678-8.349)^**^	4.734 (2.672-8.389)^**^	4.268 (2.373-7.674)^**^
*P*_for trend_	<0.001	<0.001	<0.001	<0.001

Model 1: Unadjusted.

Model 2: age, sex, marriage status, education level, occupation, total family income.

Model 3: age, sex, marriage status, education level, occupation, total family income, smoking status, drinking status, PA level, Night sleep duration; Dash score.

Model 4: age, sex, marriage status, education level, occupation, total family income, smoking status, drinking status, PA level, Night sleep duration; Dash score, hypertension, diabetes, overweight/obesity, central obesity.

DASH. dietary approaches to stop hypertension; RC. remnant cholesterol; PA. physical activity.

^*^. *P*<0.05; ^**^.*P*<0.01.

### The dose-response association between RC and HUA by restricted cubic spline

**Figure 3:**
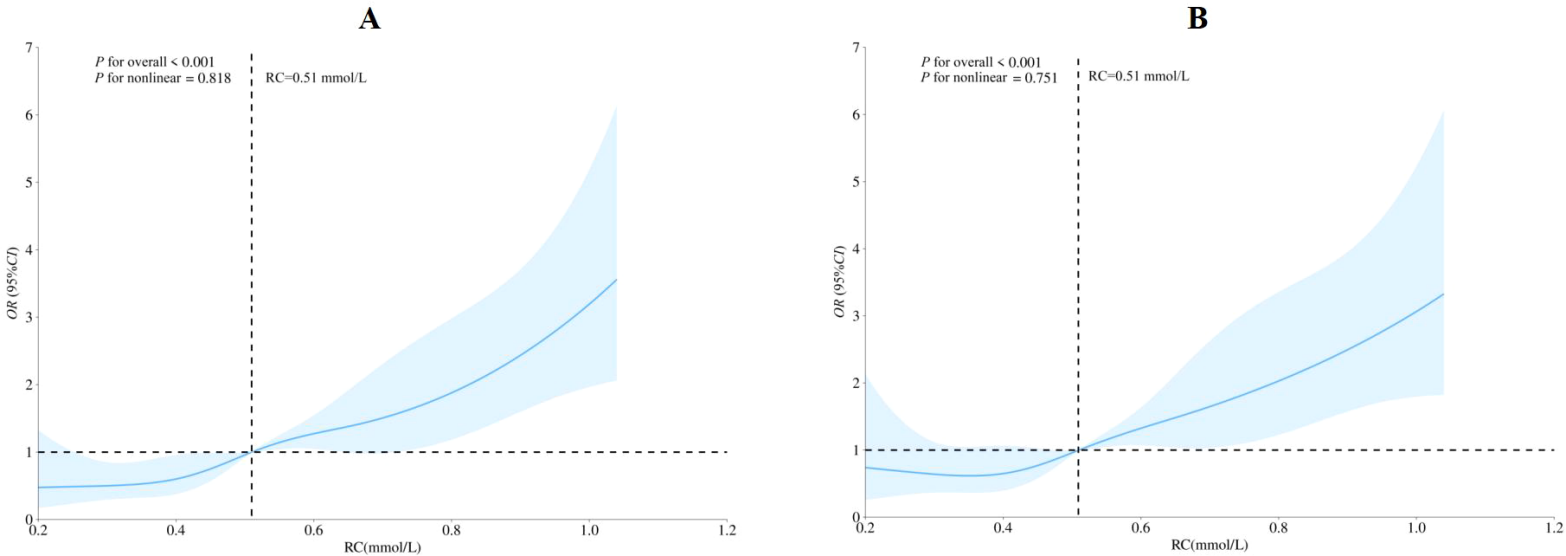
Analysis of restricted cubic spline for remnant cholesterol and hyperuricemia. **(A)**unadjusted; **(B)**adjusted for age, sex, education level, marriage status,occupation, total family income, smoking status, alcohol status, physical activity level, night sleep duration, DASH score, hypertension, diabetes, overweight and obesity, central obesity. DASH. dietary approaches to stop hypertension; CI. Confidence Interval; OR. odds ratio; RC. remnant cholesterol.

Restricted cubic spline regression revealed that RC was positively associated with HUA by a linear model (*P*_for overall_< 0.001, *P*_for non-linear_>0.05). Relative to the medium RC value of 0.51 mmol/L (*OR* = 1), the ORs of HUA exceeded unity at all higher RC levels (**Figure 3**).

### Sensitivity analyses

**Figure 4:**
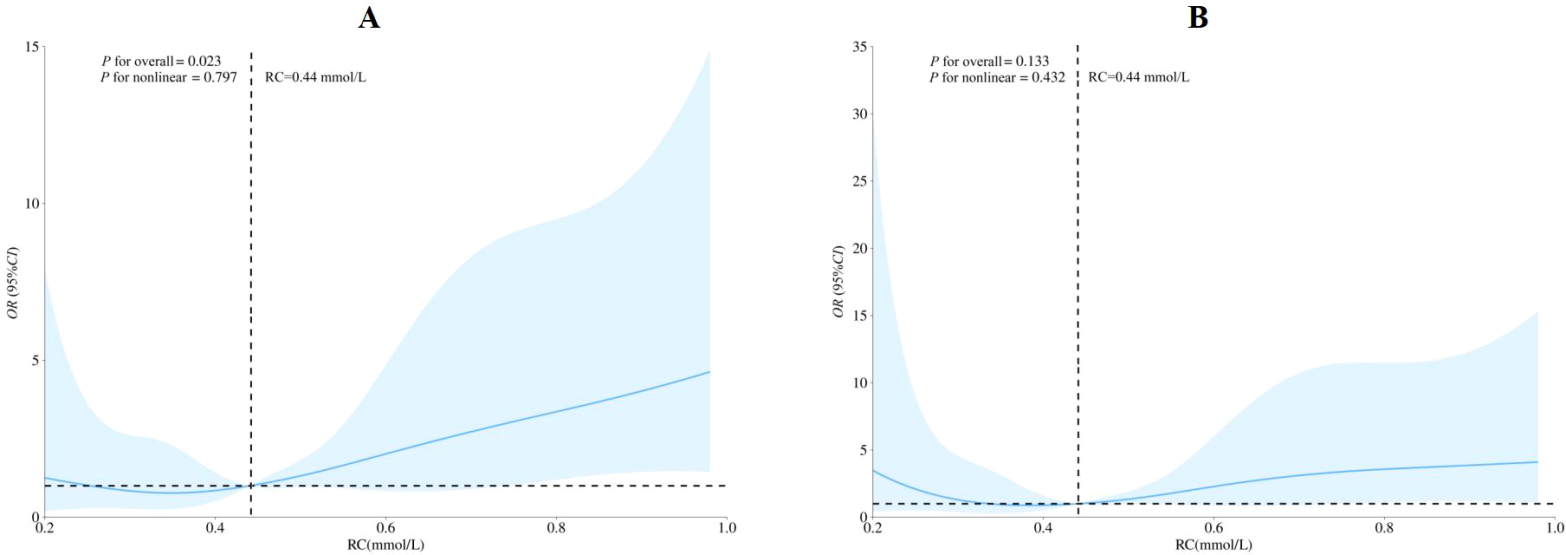
Analysis of restricted cubic spline for remnant cholesterol and hyperuricemia after excluding participants with hypertension, diabetes, overweight/obesity, central obesity. **(A) **unadjusted; **(B)** adjusted for age, sex, education level, marriage status,occupation, total family income, smoking status, alcohol status, physical activity level, night sleep duration, DASH score. DASH. dietary approaches to stop hypertension; CI. Confidence Interval; OR. odds ratio; RC. remnant cholesterol.

After excluding participants with hypertension, diabetes, overweight/obesity or central obesity, 713 participants remained. The graded association between RC and HUA persisted in multivariable-adjusted logistic regression, compared with the Q1, the ORs for the Q2, Q3 and Q4 were 1.770 (95%*CI*:0.643-4.876), 3.252 (95%*CI*:1.206-8.771) and 1.438 (95%*CI*:0.233-8.898), respectively (**Supplementary Table 5**). RCS again suggested a linear curve, with ORs > 1 for RC medium concentrations above 0.44 mmol/L (**Figure 4**). However, the overall test for the association between RC and HUA did not reach statistical significance in RCS.

### Subgroup analyses

Stratified logistic regression confirmed the robustness of the RC–HUA association across most subgroups (**Table 3**). In the second RC quartile (0.44-0.60 mmol/L), a significant excess risk was confined to participants aged 45-59 years (*OR* = 4.001, 95%*CI*:1.597-10.021), males (*OR* = 1.723, 95%*CI*:1.070-2.773), non-drinkers(*OR* = 2.373, 95%*CI*:1.135-4.965), participants without diabetes (*OR* = 1.732, 95%*CI*:1.077-2.785) and participants without central obesity (*OR* = 2.058, 95%*CI*:1.191–3.558). For the third quartile (0.61-0.89 mmol/L), the association was consistently positive in all strata, only participants aged 30-44 years (*OR* = 1.982, 95%*CI*:0.840-4.678, *P* = 0.118) and participants with diabetes (*OR* = 0.477, 95%*CI*:0.019-11.749, P = 0.651) showed non-significant estimates. In the highest RC category (≥0.90 mmol/L), the risk elevation was again evident across almost all subgroups, whereas participants aged 30-44 years (*OR* = 2.858, 95%*CI*:0.930-8.780, *P* = 0.067), participants with diabetes (*OR* = 0.088, 95%*CI*:0.002-4.455, *P* = 0.225) and participants without overweight/obesity (*OR* = 2.180, 95%*CI*:0.666-7.138, *P* = 0.198) did not reach statistical significance. Restricted cubic spline models reproduced the similar linear-shaped dose–response association within each subgroup (**Supplementary Figures 1**–**9**).

**Table 3:** Subgroup analysis of association between remnant cholesterol and hyperuricemia by logistic regression, [OR(95%CI)].

Subgroup	Q1	Q2		Q3		Q4	
		*OR*(95%CI)	*P*	*OR*(95%CI)	*P*	*OR*(95%CI)	*P*
Age/year							
30—44	1.000	1.606 (0.669 ~ 3.858)	0.289	1.982 (0.840 ~ 4.678)	0.118	2.858 (0.930 ~ 8.780)	0.067
45—59	1.000	4.001 (1.597 ~ 10.021)	0.003	7.394 (2.983 ~ 18.327)	<0.001	11.842 (3.786 ~ 37.040)	<0.001
60—79	1.000	0.920 (0.403 ~ 2.101)	0.843	2.207 (1.033 ~ 4.716)	0.041	3.182 (1.152 ~ 8.791)	0.026
Sex							
Males	1.000	1.723 (1.070 ~ 2.773)	0.025	2.515 (1.590 ~ 3.978)	<0.001	4.415 (2.436 ~ 8.002)	<0.001
Females	1.000	4.572 (0.515 ~ 40.585)	0.172	18.674 (2.364 ~ 147.497)	0.006	11.216 (1.032 ~ 121.877)	0.047
Smoking status							
No	1.000	1.609 (0.904 ~ 2.866)	0.106	3.065 (1.791 ~ 5.247)	<0.001	3.784 (1.852 ~ 7.732)	<0.001
Yes	1.000	2.132 (0.921 ~ 4.939)	0.077	2.896 (1.280 ~ 6.551)	0.011	5.101 (1.855 ~ 14.030)	0.002
Drinking status							
No	1.000	2.373 (1.135 ~ 4.965)	0.022	2.912 (1.413 ~ 6.003)	0.004	5.155 (1.965 ~ 13.524)	<0.001
Yes	1.000	1.596 (0.843 ~ 3.024)	0.151	3.097 (1.690 ~ 5.675)	<0.001	4.410 (2.034 ~ 9.562)	<0.001
Hypertension							
No	1.000	1.576 (0.832 ~ 2.982)	0.162	2.372 (1.280 ~ 4.395)	0.006	3.380 (1.497 ~ 7.628)	0.003
Yes	1.000	1.884 (0.923 ~ 3.848)	0.082	3.781 (1.896 ~ 7.541)	<0.001	6.215 (2.536 ~ 15.234)	<0.001
Diabetes							
No	1.000	1.732 (1.077 ~ 2.785)	0.024	2.870 (1.820 ~ 4.527)	<0.001	3.974 (2.172 ~ 7.272)	<0.001
Yes	1.000	0.965 (0.032 ~ 29.201)	0.984	0.477 (0.019 ~ 11.749)	0.651	0.088 (0.002 ~ 4.455)	0.225
Overweight/obesity							
No	1.000	1.729 (0.832 ~ 3.594)	0.142	3.289 (1.588 ~ 6.812)	0.001	2.180 (0.666 ~ 7.138)	0.198
Yes	1.000	1.709 (0.909 ~ 3.211)	0.096	2.736 (1.503 ~ 4.980)	<0.001	4.934 (2.379 ~ 10.235)	<0.001
Central obesity							
No	1.000	2.058 (1.191 ~ 3.558)	0.010	2.916 (1.678 ~ 5.067)	<0.001	3.257 (1.499 ~ 7.078)	0.003
Yes	1.000	1.268 (0.464 ~ 3.465)	0.643	3.077 (1.274 ~ 7.428)	0.012	6.372 (2.240 ~ 18.125)	<0.001

OR. odds ratio; CI. confidence interval; Q1: 0.14—0.43mmol/L; Q2: 0.44—0.60mmol/L; Q3: 0.61—0.89mmol/L; Q4: ≥0.90mmol/L.

### Mediation analysis between RC and UA mediated by IR

**Figure 5:**
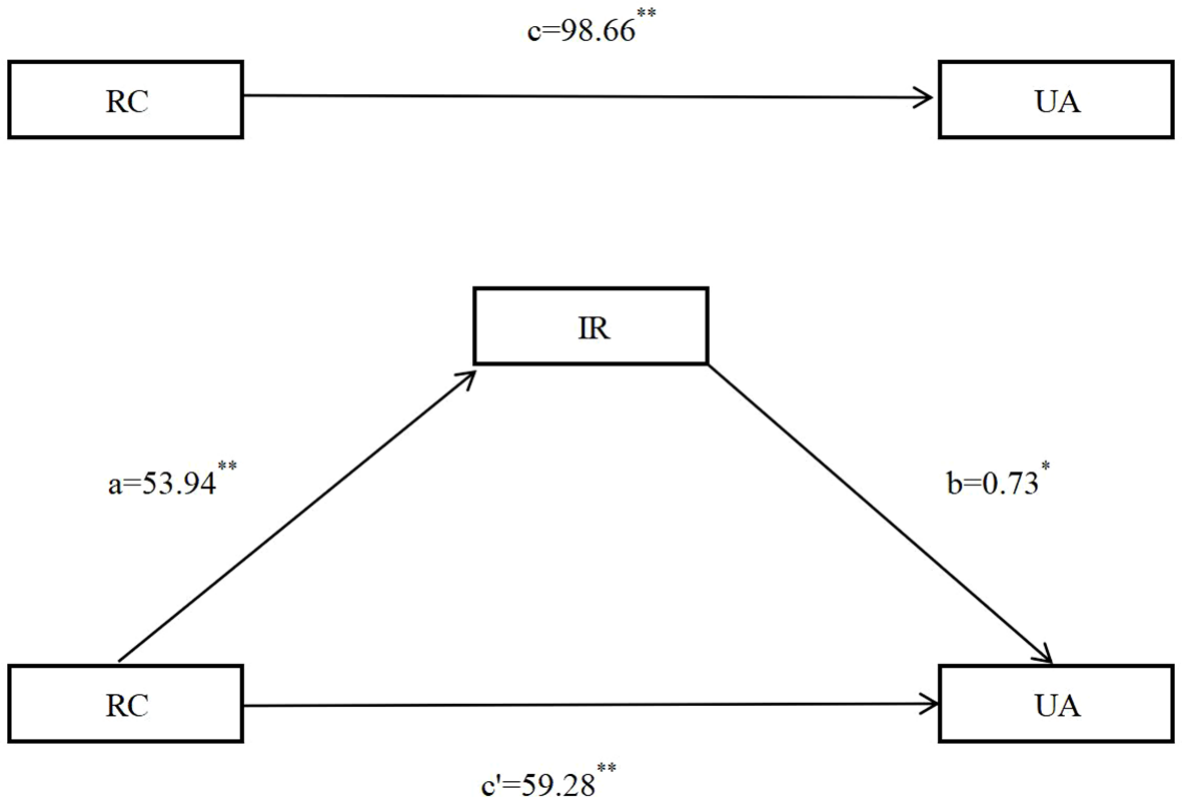
Mediation analysis between RC and UA mediated by IR after adjusting for confounding factors, including age, sex, education level, marriage status,occupation, total family income, smoking status, drinking status, physical activity level, night sleep duration, DASH score, hypertension, diabetes, overweight and obesity, central obesity. DASH. dietary approaches to stop hypertension; RC. remnant cholesterol. IR. insulin resistance; UA. uric acid. ^**^P<0.001; ^*^P<0.01.

The differences in age, sex, occupation, PA, hypertension, diabetes, HUA, overweight/obesity, central obesity, and RC across the quartiles of IR were statistically significant (*P* < 0.05) (**Table 4**). After adjusting for confounding factors, including age, sex, education level, marriage status, occupation, total family income, smoking status, drinking status, physical activity level, night sleep duration, DASH score, hypertension, diabetes, overweight and obesity, central obesity, the analysis revealed a potential mediation effect between RC and UA, the indirect effect = a×b=53.94×0.73 = 39.38, the mediation effect=39.38/98.66×100%=39.91%, indicating that IR may mediated 39.91% of the total association between RC and UA (**Table 5**; **Figure 5**).

**Table 4:** Basic characteristics of the study participants by IR [n(%)].

Variables	Total (n = 2171)	Q1 (n = 540)	Q2 (n = 477)	Q3 (n = 610)	Q4 (n = 544)	Statistic	*P*
age/year						93.670	<0.001
30—44	740 (34.09)	244 (32.97)	116 (15.68)	206 (27.84)	174 (23.51)		
45—59	858 (39.52)	193 (22.49)	219 (25.52)	262 (30.54)	184 (21.45)		
60—79	573 (26.39)	103 (17.98)	209 (36.47)	142 (24.78)	119 (20.77)		
Sex						8.880	0.031
Males	997 (45.92)	224 (22.47)	213 (21.36)	305 (30.59)	255 (25.58)		
Females	1,174 (54.08)	316 (26.92)	264 (22.49)	305 (25.98)	289 (24.62)		
Marriage status						3.758	0.289
Married/cohabiting	1,965 (90.51)	494 (25.14)	425 (21.63)	560 (28.50)	486 (24.73)		
Separated/divorced/widowed/unmarried	206 (9.49)	46 (22.33)	52 (25.24)	50 (24.27)	58 (28.16)		
Education level						9.465	0.149
Primary school or below	521 (24.00)	124 (23.80)	150 (28.79)	147 (28.21)	100 (19.19)		
Junior middle school	627 (28.88)	145 (23.13)	159 (25.36)	175 (27.91)	148 (23.60)		
High school or above	1,023 (47.12)	271 (26.49)	235 (22.97)	288 (28.15)	229 (22.39)		
Occupation						40.625	<0.001
Farmers	705 (32.47)	164 (23.26)	143 (20.28)	194 (27.52)	204 (28.94)		
Government employees	233 (10.73)	72 (30.90)	47 (20.17)	60 (25.75)	54 (23.18)		
Workers	204 (9.40)	61 (29.90)	43 (21.08)	61 (29.90)	39 (19.12)		
Sales staff	371 (17.09)	111 (29.92)	77 (20.75)	117 (31.54)	66 (17.79)		
Others	658 (30.31)	132 (20.06)	167 (25.38)	178 (27.05)	181 (27.51)		
Total family income/yuan						10.868	0.285
<20000	671 (30.91)	172 (25.63)	152 (22.65)	169 (25.19)	178 (26.53)		
20,000—59,999	790 (36.39)	196 (24.81)	165 (20.89)	235 (29.75)	194 (24.56)		
60,000—99,999	372 (17.13)	83 (22.31)	82 (22.04)	103 (27.69)	104 (27.96)		
≥100,000	338 (15.57)	89 (26.33)	78 (23.08)	103 (30.47)	68 (20.12)		
Smoking status						3.615	0.306
No	1,793 (82.59)	459 (25.60)	396 (22.09)	496 (27.66)	442 (24.65)		
Yes	378 (17.41)	81 (21.43)	81 (21.43)	114 (30.16)	102 (26.98)		
Drinking status						2.789	0.425
No	1,085 (49.98)	286 (26.36)	238 (21.94)	296 (27.28)	265 (24.42)		
Yes	1,086 (50.02)	254 (23.39)	239 (22.01)	314 (28.91)	279 (25.69)		
PA level						33.539	<0.001
Low	297 (13.71)	55 (18.52)	70 (23.57)	75 (25.25)	97 (32.66)		
Moderate	337 (15.55)	60 (17.80)	81 (24.04)	94 (27.89)	102 (30.27)		
Vigorous	1,533 (70.74)	424 (27.66)	325 (21.20)	440 (28.70)	344 (22.44)		
Night sleep duration						4.808	0.569
Insufficient	559 (25.75)	127 (22.72)	149 (26.65)	162 (28.98)	121 (21.65)		
Sufficient	1,392 (64.12)	354 (25.43)	342 (24.57)	395 (28.38)	301 (21.62)		
Excessive	220 (10.13)	59 (26.82)	53 (24.09)	53 (24.09)	55 (25.00)		
Dash score						5.253	0.512
≤20	725 (33.39)	175 (24.14)	187 (25.79)	208 (28.69)	155 (21.38)		
21—24	628 (28.93)	143 (22.77)	161 (25.64)	186 (29.62)	138 (21.97)		
≥25	818 (37.68)	222 (27.14)	196 (23.96)	216 (26.41)	184 (22.49)		
Hypertension						95.274	<0.001
No	1,398 (64.39)	420 (30.04)	319 (22.82)	389 (27.83)	270 (19.31)		
Yes	773 (35.61)	120 (15.52)	158 (20.44)	221 (28.59)	274 (35.45)		
Diabetes						257.398	<0.001
No	2,008 (92.49)	536 (26.69)	471 (23.46)	582 (28.98)	419 (20.87)		
Yes	163 (7.51)	4 (2.45)	6 (3.68)	28 (17.18)	125 (76.69)		
Hyperuricemia						55.349	<0.001
No	1,974 (90.93)	520 (26.34)	447 (22.64)	550 (27.86)	457 (23.15)		
Yes	197 (9.07)	20 (10.15)	30 (15.23)	60 (30.46)	87 (44.16)		
Overweight/obesity						407.662	<0.001
No	1,053 (48.50)	411 (39.03)	286 (27.16)	261 (24.79)	95 (9.02)		
Yes	1,118 (51.50)	129 (11.54)	191 (17.08)	349 (31.22)	449 (40.16)		
Central obesity						235.238	<0.001
No	1,647 (75.86)	494 (29.99)	394 (23.92)	469 (28.48)	290 (17.61)		
Yes	524 (24.14)	46 (8.78)	83 (15.84)	141 (26.91)	254 (48.47)		
RC							<0.001
0.14—0.43	741 (34.13)	520 (70.18)	29 (3.91)	185 (24.97)	7 (0.94)	2,221.007	
0.44—0.60	649 (29.89)	20 (3.08)	293 (45.15)	274 (42.22)	62 (9.55)		
0.61—0.89	629 (28.97)	0 (0.00)	280 (44.52)	18 (2.86)	331 (52.62)		
≥0.90	152 (7.00)	0 (0.00)	8 (5.26)	0 (0.00)	144 (94.74)		

DASH. dietary approaches to stop hypertension; RC. remnant cholesterol; PA. physical activity; IR. insulin resistance; Q1:<18.14; Q2:18.14—25.63; Q3:25.64—35.81; Q4:≥35.82. Statistic. analysed by chi square test.

**Table 5:** Mediation analysis between RC and UA.

Path		β	S.E	*t*	*P*	*β* (95%*CI*)
RC→IR	a	53.94	0.697	77.371	<0.001	53.94 (52.57-55.31)
RC→UA	c’	59.28	15.808	3.750	<0.001	59.28 (28.29-90.26)
IR→UA	b	0.73	0.251	2.885	0.004	0.73 (0.23-1.22)
RC→UA	c	98.66	8.166	12.044	<0.001	98.66 (82.35- 114.36)

S.E. standard error; CI. confidence interval; RC. remnant cholesterol. IR. insulin resistance; UA. uric acid.

## Discussion

This study is the first to systematically evaluate the independent association, dose-response association and underlying mechanisms between RC and UA, as well as HUA risk, in a natural Chinese adult population with normal lipid profiles in Southwestern China. The key findings are summarized as follows: (1) RC exhibited a strong, positive, dose-dependent, and linear-shaped association with HUA, independent of established risk factors; (2) this association persisted even after excluding individuals with metabolic abnormalities such as hypertension, diabetes, and obesity, indicating that RC is an upstream determinant rather than a downstream epiphenomenon of metabolic syndrome; (3) participants aged 45–59 years, males, non-smokers, non-drinkers, and those without hypertension, hyperglycemia, overweight/obesity, or central obesity were more susceptible to elevated RC; and (4) IR mediated approximately 40% of the association between RC and UA, suggesting that the RC → IR → UA pathway may represent a clinically actionable target. However, differences in study design, population characteristics (e.g., age, sex, ethnicity, and baseline metabolic status), variations in RC calculation methods, and insufficient control for confounders (such as renal function and dietary purine intake) may influence the observed association between RC and HUA.

RC represents the total cholesterol concentration excluding LDL-C and HDL-C. Under fasting conditions, it primarily comprises very-low-density lipoprotein cholesterol (VLDL-C) and intermediate-density lipoprotein cholesterol (IDL-C), while in the non-fasting state, it also includes chylomicrons (39, 40). RC has been extensively studied in the context of lipid metabolism disorders and is recognized as a pro-atherogenic lipoprotein. Notably, substantial evidence has established elevated RC as a significant contributor to the pathogenesis of cardiovascular and metabolic diseases, including atherosclerosis, stroke, hypertension, and diabetes (41–44). Compelling evidence has further shown that RC serves as an independent predictor not only for cardiovascular diseases but also for a range of metabolic conditions (45, 46). However, evidence regarding the association between RC and HUA remains limited, although prior studies suggest a positive correlation between RC and HUA risk (4, 16, 47) comprising dyslipidaemia participants leave unresolved whether the association exists in normolipidemic individuals.

Previous studies have predominantly examined the association of triglycerides (TG) or triglyceride-rich lipoproteins (TRLs) with gout/HUA (48, 49). In contrast, direct epidemiological data quantifying RC, defined as the cholesterol content within chylomicron and VLDL remnants, remain exceedingly scarce. Although RC is strongly correlated with TG, it more accurately reflects the atherogenic cholesterol burden carried by remnant particles and is less influenced by fasting status, thereby providing greater stability as a biomarker. We identified a strong and graded association between increasing quartiles of RC and the prevalence of HUA within a normolipidemic population, with rates increasing from 4.45% in the lowest quartile to 19.08% in the highest. Even after comprehensive multivariate adjustment, participants in the highest RC quartile had 4.268 times the odds of having HUA compared with those in the lowest quartile, confirming that elevated RC is robustly associated with HUA risk. These findings partially corroborate previous studies (4, 16). RCS revealed a steep, nearly linear increase in HUA risk once RC exceeded 0.51 mmol/L, suggesting that this concentration may represent a clinically relevant threshold for incident HUA in Chinese adults, however, longitudinal or prospective validation is warranted.

RC identifies a clinically distinct subset of hyperuricemia individuals who are metabolically “invisible” to conventional risk algorithms. Clinically, for individuals without typical risk factors but with RC > 0.51 mmol/L, systematic UA screening and aggressive lifestyle modification are recommended. Among patients with established HUA and concomitantly elevated RC, integrating urate-lowering therapy with fibrate-based, ω-3 fatty acid, or emerging ANGPTL3-targeted regimens to reduce remnant lipoproteins may provide dual cardiometabolic benefits. After excluding participants with hypertension, diabetes, overweight/obesity, and central obesity, sensitivity analyses confirmed a persistent positive association between RC and HUA risk, underscoring the robustness of this association. However, statistical significance was confined to the RC range of 0.61–0.89 mmol/L, likely due to reduced statistical power following these exclusions. RCS further revealed a steep increase in HUA risk beginning at RC ≥ 0.44 mmol/L, suggesting that HUA risk in relatively healthier populations may be more sensitive to variations in RC levels.

Our study revealed significant sex-specific heterogeneity in the association between RC and HUA, with a markedly stronger association observed in females than in males. This finding is consistent with the report by Zhou (16) et al., and a potential explanation for this finding involves the established role of estrogen in the regulation of lipid and glucose metabolism in females (50). The decline in estrogen levels following menopause may predispose females to dysregulated lipid metabolism, thereby increasing their susceptibility to HUA (16). Among middle-aged and older adults, insufficient physical activity is postulated as a key behavioral pathway promoting adiposity, which in turn drives an increase in circulating RC levels (51). Our analyses revealed a significantly stronger association between RC and HUA risk among participants aged 45–59 years, suggesting a window of particularly vulnerability during this life stage. This demographic corresponds to a phase of accelerated decline in insulin sensitivity, characterized by increased hepatic VLDL secretion coupled with impaired remnant clearance, collectively creating a distinct metabolic window.

The RC–HUA association was more pronounced in hypertensive versus normotensive participants, plausibly because hypertension lowered UA clearance by reducing glomerular filtration and intensifying tubular sodium-urate exchange (52). Moreover, the hypertensive milieu exhibited a higher insulin-resistance burden, and hyperinsulinaemia suppressed the renal urate transporter URAT1 (53), further curtailing excretion. However, subgroup analyses revealed that the positive association between RC and HUA remained statistically significant even among normotensive participants, indicating that this association is independent of blood pressure status. It is well established that certain glucose-lowering drugs can stimulate uric acid production and secretion (54). Analyses restricted to participants without diabetes similarly revealed a monotonic, statistically significant increase in hyperuricemia risk across rising remnant cholesterol concentrations, corroborating that the RC–HUA association is independent of glucose homeostasis. Obesity is frequently accompanied by dyslipidemia, which can influence circulating RC levels (55). Adipose-derived free fatty acids (FFAs) flood the liver in obese participants, were re-esterified to triglycerides, and packaged into very-low-density lipoprotein (52), directly raising remnant cholesterol. Concurrently, chronic low-grade inflammation (elevated CRP and WBC) suppressed membrane localization of renal urate efflux transporters, curtailing uric acid elimination (56). Subgroup analyses further revealed that elevated RC levels remained significantly associated with an increased risk of HUA among participants without overweight/obesity or central obesity, indicating that this association is not mediated by excess body fat. These findings highlight the clinical importance of targeting elevated RC for intervention, regardless of overweight status.

An intriguing observation emerged from our RCS analysis: the threshold of RC associated with increased HUA risk was consistently lower in metabolically healthy participants (with normal blood pressure, glucose, body weight, and waist circumference) compared to those with metabolic abnormalities. This pattern suggests greater metabolic sensitivity to RC fluctuations in healthy individuals, where even mild elevations in RC may disrupt uric acid homeostasis and precipitate hyperuricemia. Conversely, individuals with conditions such as overweight/obesity, hypertension, or hyperglycemia typically present with baseline metabolic disturbances, including insulin resistance and chronic low-grade inflammation, both known to elevate uric acid. This underlying metabolic dysregulation may obscure the independent effect of RC on HUA risk. This suggests a potential threshold effect, wherein a higher RC burden is necessary to exert a statistically discernible additive effect on HUA risk within an already compromised metabolic milieu (52, 57).

Based on established studies regarding the interplay between IR and dyslipidemia (58), we specifically quantified the mediating role of IR. Our analyses confirmed that IR significantly mediates the positive association between RC and UA levels, accounting for a potential mediation proportion of 39.91%. This suggests that RC acts as a core driver of hyperuricemia, while IR may function primarily as a “metabolic amplifier” partially transmitting the effect of RC. Consequently, therapeutic strategies focused solely on improving insulin sensitivity are unlikely to eliminate the excess UA risk imposed by elevated RC. These data establish lowering RC as a therapeutic priority. First-line interventions, including fibrate-based regimens and intensive lifestyle modification, should be initiated promptly in all patients with elevated RC. When IR coexists, combination therapy simultaneously addressing multiple components of metabolic syndrome is indicated. Sodium–glucose cotransporter-2 (SGLT2) inhibitors are especially attractive in this context, as they reduce RC-rich lipoproteins, enhance insulin sensitivity, and augment uric acid excretion, thereby delivering integrated cardiometabolic and urate-lowering benefits. Our findings support incorporating RC into personalized risk algorithms, classifying individuals with RC above the 75th percentile as “high-RC” risk individuals who would otherwise be missed by conventional diabetes-centric screening. For these patients, systematic uric acid surveillance should be initiated even in the absence of IR reaching the threshold for diabetes.

Mechanistically, elevated RC fosters UA retention via a coordinated “liver–kidney–peripheral” axis (1): Hepatic limb: RC particles are enriched in apolipoprotein C-III (apoC-III), which antagonises both lipoprotein lipase and LDL-receptor activity, prolonging the circulatory residence of triglyceride-rich lipoproteins and their UA cargo. Simultaneously, apoC-III activates TLR4/NF-κB signalling, transcriptionally up-regulating hepatic xanthine oxidase (XOD) and thereby accelerating UA synthesis (59). (2) Renal limb: Because of their large diameter, remnant lipoproteins are efficiently filtered and avidly taken up by glomerular endothelial cells, where they evoke localized oxidative stress and NF-κB-driven inflammation. The ensuing endothelial dysfunction and vasoconstriction reduce renal plasma flow and depress tubular uric-acid secretion, manifesting as a fall in fractional excretion of UA (FEUA) and a concomitant rise in serum UA (60). (3) Peripheral Pathway: RC-induced insulin resistance (IR) elevates plasma insulin levels, which in turn stimulate the urate transporter URAT1 and suppress renal uric acid excretion (61), thereby further amplifying hyperuricemia effect (61). Mediation analysis confirmed that IR accounts for 40% of potential mediation effect for the observed association, which aligns remarkably well with the proposed mechanistic pathway.

Our study highlights RC as an overlooked metabolic risk factor. While traditionally recognized as a risk factor for atherosclerosis and cardiovascular diseases, our findings demonstrate a positive association between RC and HUA, thereby expanding the spectrum of its pathological roles. This suggests that RC not only contributes to dyslipidemia but may also indirectly drive uric acid elevation via insulin resistance-mediated pathways. Given that RC is simple to calculate and cost-effective to measure, it holds significant potential for widespread clinical adoption. Our study indicates that elevated RC may serve as an independent predictor of HUA, even in healthy individuals, and may be utilized as a novel tool in routine health examinations to identify populations at risk of hyperuricemia, particularly in the context of early screening for metabolic syndrome.

The current study has important implications for clinical practice. Current lipid management strategies primarily focus on LDL-C, while our findings indicate that elevated RC is an independent risk factor for hyperuricemia even among clinically healthy individuals, particularly in the early stages of metabolic syndrome. Therefore, lipid management should not be limited to LDL-C but should incorporate RC into routine assessment to achieve more comprehensive cardiometabolic risk control. Traditional interventions for hyperuricemia mainly target purine intake, uric acid excretion, and renal function regulation. However, this study suggests that controlling RC levels and improving insulin resistance may provide new intervention targets for hyperuricemia prevention and treatment, particularly suitable for apparently healthy individuals with elevated RC but no typical metabolic abnormalities. Furthermore, incorporating RC into hyperuricemia risk prediction models can significantly enhance the early identification capability in the general population, addressing the blind spots of traditional models that rely on BMI and renal function indicators, thereby offering new strategies for the early prevention and control of hyperuricemia.

This study has several notable strengths. First, it is based on a large community-based study, enhancing the generalizability of the findings. Second, RC was directly quantified using a standardized enzymatic assay, minimizing misclassification and avoiding postprandial bias. Third, the consistent positive association between RC and HUA risk observed in this normolipidemic population from rural Western China, together with the confirmation that elevated RC is an independent risk factor for HUA, was robust across multiple adjusted models and sensitivity analyses. Importantly, this association remained significant even among participants with normal blood pressure, glucose, body weight, and waist circumference. Importantly, this study is the first to quantify the mediating effect of IR in the RC-HUA pathway, revealing that it accounts for approximately 40% of the total association, providing a quantitative basis for understanding targeted interventions. Despite these strengths, several limitations warrant consideration. First, the cross-sectional design precludes definitive causal inference between RC and HUA, necessitating validation in prospective cohorts. Second, the mechanistic pathway remains partially elucidated, as we did not measure key biomarkers such as xanthine oxidase (XOD) activity, fractional excretion of uric acid (FEUA), or URAT1 gene polymorphisms. Third, potential residual confounding (e.g., from dietary purine intake or subtle differences in renal function) cannot be fully excluded. Third, we were unable to obtain and adjust for several important confounding factors. Specifically, dietary purine intake is a key determinant of serum uric acid levels. However, our subgroup analyses based on DASH score consistently revealed a similar association between RC and HUA across different subgroups (**Supplementary Figures 9**), which may partially mitigate concerns regarding this unmeasured confounder. In contrast, renal function parameters (such as estimated glomerular filtration rate or urinary protein) serve as major regulators of uric acid excretion. The lack of data on these variables may introduce residual confounding in our analysis. Fourth, our study was conducted in Southwestern China with a sample size of approximately 3,000 participants, which may limit the generalizability of our findings to other populations. Nevertheless, the identification of RC as a potential indicator for HUA screening and prevention offers a novel approach for early risk assessment and intervention. We believe this contribution provides valuable insights and a fresh direction for future public health strategies aimed at HUA control. Finally, the sample size in the “healthy” subgroup, after excluding individuals with multiple metabolic abnormalities was reduced, resulting in wider confidence intervals for the odds ratio in the highest RC quartile, which should therefore be interpreted with caution. Based on our findings, we propose the following future research directions: initiating multicenter prospective cohorts to assess the 5–10-year cumulative risk of HUA and gout incidence using the 0.51 mmol/L RC threshold; conducting intervention trials to compare the efficacy of combined therapy (e.g., fenofibrate, eicosapentaenoic acid, and allopurinol versus allopurinol monotherapy on UA levels and gout attack rates; and utilizing humanized liver–kidney organ-on-a-chip models to dissect the direct effects of remnant cholesterol particle size and the cholesterol/apoc-III ratio on URAT1/ABCG2 transporter function, thereby providing experimental evidence for future targeted therapies.

## Conclusion

Remnant cholesterol may emerge as an independent, dose-dependent, and readily modifiable determinant of incident hyperuricemia in Chinese adults, with approximately 40% of potential effect mediated through insulin resistance. Direct, fasting-independent enzymatic quantification and well-defined dose–response thresholds make RC an ideal candidate for inclusion in routine metabolic panels and present as a hypothesis for future longitudinal research.

## Data Availability

The original contributions presented in the study are included in the article/**Supplementary Material**. Further inquiries can be directed to the corresponding author.
