# Supplementary material for: Remnant cholesterol: an independent, dose-dependent risk factor for hyperuricemia in a normolipidemic chinese population
Source: Front Endocrinol (Lausanne). 2026 Jan 12;16:1718817. doi: 10.3389/fendo.2025.1718817 (PMC12832488; doi:10.3389/fendo.2025.1718817)
Supplement: Supplementary file 11 [file Table1.docx]

Supplementary Table 1. Unadjusted Model 1: association between residual cholesterol and hyperuricemia by logistic regression

| Variables | β | S.E | Z | *P* | OR (95%CI) |
| --- | --- | --- | --- | --- | --- |
|  |  |  |  |  |  |
| RC |  |  |  |  |  |
| 0.14—0.43 |  |  |  |  | 1.000 (Reference) |
| 0.44—0.60 | 0.666 | 0.228 | 2.925 | **0.003** | 1.947 (1.246-3.043) |
| 0.61—0.89 | 1.154 | 0.214 | 5.388 | **<0.001** | 3.171 (2.084-4.826) |
| ≥0.90 | 1.621 | 0.273 | 5.946 | **<0.001** | 5.058 (2.965-8.631) |
| OR: Odds Ratio, CI: Confidence Interval | | | | | |
