# Supplementary material for: Remnant cholesterol: an independent, dose-dependent risk factor for hyperuricemia in a normolipidemic chinese population
Source: Front Endocrinol (Lausanne). 2026 Jan 12;16:1718817. doi: 10.3389/fendo.2025.1718817 (PMC12832488; doi:10.3389/fendo.2025.1718817)
Supplement: Supplementary file 12 [file Table2.docx]

Supplementary Table 2. Adjusted Model 2: association between residual cholesterol and hyperuricemia by logistic regression

| Variables | β | S.E | Z | *P* | OR (95%CI) |
| --- | --- | --- | --- | --- | --- |
|  |  |  |  |  |  |
| Intercept | -2.418 | 0.324 | -7.469 | **<0.001** | 0.089 (0.047-0.168) |
| RC |  |  |  |  |  |
| 0.14—0.43 |  |  |  |  | 1.000 (Reference) |
| 0.44—0.60 | 0.617 | 0.235 | 2.629 | **0.009** | 1.854 (1.170-2.937) |
| 0.61—0.89 | 1.161 | 0.223 | 5.208 | **<0.001** | 3.193 (2.063-4.943) |
| ≥0.90 | 1.554 | 0.290 | 5.357 | **<0.001** | 4.729 (2.678-8.349) |
| age/year |  |  |  |  |  |
| 30—44 |  |  |  |  | 1.000 (Reference) |
| 45—59 | 0.059 | 0.202 | 0.289 | 0.772 | 1.060 (0.713-1.577) |
| 60—79 | 0.269 | 0.238 | 1.130 | 0.258 | 1.309 (0.821-2.088) |
| Sex |  |  |  |  |  |
| Males |  |  |  |  | 1.000 (Reference) |
| Females | -2.210 | 0.220 | -10.047 | **<0.001** | 0.110 (0.071-0.169) |
| Marriage status |  |  |  |  |  |
| Married/cohabiting |  |  |  |  | 1.000 (Reference) |
| Separated/divorced/widowed/unmarried | -0.019 | 0.306 | -0.062 | 0.950 | 0.981 (0.539-1.787) |
| Education level |  |  |  |  |  |
| Primary school or below |  |  |  |  | 1.000 (Reference) |
| Junior middle school | -0.065 | 0.224 | -0.291 | 0.771 | 0.937 (0.605-1.452) |
| High school or above | -0.295 | 0.195 | -1.510 | 0.131 | 0.745 (0.508-1.092) |
| Occupation |  |  |  |  |  |
| Farmers |  |  |  |  | 1.000 (Reference) |
| Government employees | 0.470 | 0.297 | 1.584 | 0.113 | 1.600 (0.895-2.861) |
| Workers | 0.265 | 0.284 | 0.935 | 0.350 | 1.304 (0.747-2.275) |
| Sales staff | 0.278 | 0.281 | 0.992 | 0.321 | 1.321 (0.762-2.290) |
| Others | 0.266 | 0.213 | 1.252 | 0.211 | 1.305 (0.860-1.981) |
| Total family income/yuan |  |  |  |  |  |
| ＜20000 |  |  |  |  | 1.000 (Reference) |
| 20,000—59,999 | -0.118 | 0.202 | -0.584 | 0.559 | 0.889 (0.598-1.321) |
| 60,000—99,999 | -0.047 | 0.244 | -0.194 | 0.846 | 0.954 (0.591-1.540) |
| ≥100,000 | -0.059 | 0.263 | -0.225 | 0.822 | 0.942 (0.563-1.579) |
| OR. Odds Ratio, CI.Confidence Interval; RC. remnant cholesterol | | | | | |
