# Supplementary material for: Remnant cholesterol: an independent, dose-dependent risk factor for hyperuricemia in a normolipidemic chinese population
Source: Front Endocrinol (Lausanne). 2026 Jan 12;16:1718817. doi: 10.3389/fendo.2025.1718817 (PMC12832488; doi:10.3389/fendo.2025.1718817)
Supplement: Supplementary file 13 [file Table3.docx]

Supplementary Table 3. Adjusted Model 3: association between residual cholesterol and hyperuricemia by logistic regression

| Variables | β | S.E | Z | *P* | OR (95%CI) |
| --- | --- | --- | --- | --- | --- |
|  |  |  |  |  |  |
| Intercept | -2.082 | 0.450 | -4.631 | **<0.001** | 0.125 (0.052-0.301) |
| RC |  |  |  |  |  |
| 0.14—0.43 |  |  |  |  | 1.000 (Reference) |
| 0.44—0.60 | 0.621 | 0.237 | 2.619 | **0.009** | 1.860 (1.169-2.960) |
| 0.61—0.89 | 1.186 | 0.224 | 5.282 | **<0.001** | 3.273 (2.108-5.081) |
| ≥0.90 | 1.555 | 0.292 | 5.327 | **<0.001** | 4.734 (2.672-8.389) |
| age/year |  |  |  |  |  |
| 30—44 |  |  |  |  | 1.000 (Reference) |
| 45—59 | 0.042 | 0.206 | 0.205 | 0.837 | 1.043 (0.697-1.561) |
| 60—79 | 0.218 | 0.251 | 0.867 | 0.386 | 1.243 (0.760-2.033) |
| Sex |  |  |  |  |  |
| Males |  |  |  |  | 1.000 (Reference) |
| Females | -2.246 | 0.234 | -9.587 | **<0.001** | 0.106 (0.067-0.167) |
| Marriage status |  |  |  |  |  |
| Married/cohabiting |  |  |  |  | 1.000 (Reference) |
| Separated/divorced/widowed/unmarried | 0.006 | 0.306 | 0.020 | 0.984 | 1.006 (0.552-1.834) |
| Education level |  |  |  |  |  |
| Primary school or below |  |  |  |  | 1.000 (Reference) |
| Junior middle school | -0.094 | 0.226 | -0.418 | 0.676 | 0.910 (0.585-1.416) |
| High school or above | -0.291 | 0.197 | -1.475 | 0.140 | 0.748 (0.508-1.100) |
| Occupation |  |  |  |  |  |
| Farmers |  |  |  |  | 1.000 (Reference) |
| Government employees | 0.490 | 0.300 | 1.632 | 0.103 | 1.633 (0.906-2.942) |
| Workers | 0.263 | 0.286 | 0.918 | 0.358 | 1.301 (0.742-2.279) |
| Sales staff | 0.271 | 0.283 | 0.959 | 0.338 | 1.312 (0.753-2.284) |
| Others | 0.234 | 0.226 | 1.036 | 0.300 | 1.263 (0.812-1.966) |
| Total family income/yuan |  |  |  |  |  |
| ＜20000 |  |  |  |  | 1.000 (Reference) |
| 20,000—59,999 | -0.061 | 0.205 | -0.299 | 0.765 | 0.941 (0.630-1.405) |
| 60,000—99,999 | -0.025 | 0.249 | -0.101 | 0.920 | 0.975 (0.599-1.588) |
| ≥100,000 | -0.008 | 0.268 | -0.029 | 0.977 | 0.992 (0.587-1.677) |
| Smoking status |  |  |  |  |  |
| No |  |  |  |  | 1.000 (Reference) |
| Yes | -0.133 | 0.185 | -0.720 | 0.471 | 0.875 (0.610-1.257) |
| Dringking status |  |  |  |  |  |
| No |  |  |  |  | 1.000 (Reference) |
| Yes | 0.078 | 0.173 | 0.452 | 0.652 | 1.081 (0.770-1.518) |
| PA level |  |  |  |  |  |
| Low |  |  |  |  | 1.000 (Reference) |
| Moderate | -0.159 | 0.302 | -0.528 | 0.598 | 0.853 (0.472-1.541) |
| Vigorous | -0.131 | 0.263 | -0.498 | 0.619 | 0.877 (0.524-1.469) |
| Night sleep duration, |  |  |  |  |  |
| Insufficient |  |  |  |  | 1.000 (Reference) |
| Sufficient | -0.321 | 0.184 | -1.742 | 0.081 | 0.726 (0.506-1.041) |
| Excessive | 0.137 | 0.278 | 0.494 | 0.621 | 1.147 (0.665-1.977) |
| Dash score |  |  |  |  |  |
| ≤20 |  |  |  |  | 1.000 (Reference) |
| 21—24 | -0.128 | 0.201 | -0.636 | 0.525 | 0.880 (0.594-1.305) |
| ≥25 | -0.033 | 0.194 | -0.167 | 0.867 | 0.968 (0.661-1.417) |
| OR. Odds Ratio, CI. Confidence Interval; DASH. dietary approaches to stop hypertension; RC. remnant cholesterol; PA. physical activity | | | | | |
