# Supplementary material for: Remnant cholesterol: an independent, dose-dependent risk factor for hyperuricemia in a normolipidemic chinese population
Source: Front Endocrinol (Lausanne). 2026 Jan 12;16:1718817. doi: 10.3389/fendo.2025.1718817 (PMC12832488; doi:10.3389/fendo.2025.1718817)
Supplement: Supplementary file 14 [file Table4.docx]

Supplementary Table 4. Adjusted Model 4: association between residual cholesterol and hyperuricemia by logistic regression

| Variables | β | S.E | Z | *P* | OR (95%CI) |
| --- | --- | --- | --- | --- | --- |
|  |  |  |  |  |  |
| Intercept | -2.394 | 0.462 | -5.179 | **<.001** | 0.091 (0.037-0.226) |
| RC |  |  |  |  |  |
| 0.14—0.43 |  |  |  |  | 1.000 (Reference) |
| 0.44—0.60 | 0.561 | 0.241 | 2.331 | **0.020** | 1.753 (1.093-2.809) |
| 0.61—0.89 | 1.065 | 0.231 | 4.614 | **<0.001** | 2.900 (1.845-4.558) |
| ≥0.90 | 1.451 | 0.299 | 4.847 | **<0.001** | 4.268 (2.373-7.674) |
| age/year |  |  |  |  |  |
| 30—44 |  |  |  |  | 1.000 (Reference) |
| 45—59 | -0.093 | 0.215 | -0.435 | 0.664 | 0.911 (0.598-1.387) |
| 60—79 | -0.007 | 0.271 | -0.026 | 0.979 | 0.993 (0.584-1.688) |
| Sex |  |  |  |  |  |
| Males |  |  |  |  | 1.000 (Reference) |
| Females | -2.269 | 0.238 | -9.550 | **<0.001** | 0.103 (0.065-0.165) |
| Marriage status |  |  |  |  |  |
| Married/cohabiting |  |  |  |  | 1.000 (Reference) |
| Separated/divorced/widowed/unmarried | 0.014 | 0.311 | 0.047 | 0.963 | 1.015 (0.552-1.866) |
| Education level |  |  |  |  |  |
| Primary school or below |  |  |  |  | 1.000 (Reference) |
| Junior middle school | -0.156 | 0.229 | -0.679 | 0.497 | 0.856 (0.546-1.341) |
| High school or above | -0.307 | 0.201 | -1.530 | 0.126 | 0.736 (0.496-1.090) |
| Occupation |  |  |  |  |  |
| Farmers |  |  |  |  | 1.000 (Reference) |
| Government employees | 0.479 | 0.305 | 1.572 | 0.116 | 1.614 (0.889-2.932) |
| Workers | 0.282 | 0.290 | 0.973 | 0.331 | 1.326 (0.751-2.340) |
| Sales staff | 0.255 | 0.288 | 0.887 | 0.375 | 1.291 (0.734-2.270) |
| Others | 0.309 | 0.228 | 1.354 | 0.176 | 1.363 (0.871-2.132) |
| Total family income/yuan |  |  |  |  |  |
| ＜20000 |  |  |  |  | 1.000 (Reference) |
| 20,000—59,999 | -0.075 | 0.209 | -0.357 | 0.721 | 0.928 (0.616-1.398) |
| 60,000—99,999 | 0.010 | 0.254 | 0.041 | 0.968 | 1.010 (0.614-1.661) |
| ≥100,000 | 0.045 | 0.272 | 0.164 | 0.870 | 1.046 (0.614-1.782) |
| Smoking status |  |  |  |  |  |
| No |  |  |  |  | 1.000 (Reference) |
| Yes | -0.116 | 0.189 | -0.614 | 0.539 | 0.891 (0.615-1.289) |
| Dringking status |  |  |  |  |  |
| No |  |  |  |  | 1.000 (Reference) |
| Yes | -0.012 | 0.176 | -0.067 | 0.947 | 0.988 (0.700-1.395) |
| PA level |  |  |  |  |  |
| Low |  |  |  |  | 1.000 (Reference) |
| Moderate | -0.080 | 0.306 | -0.263 | 0.792 | 0.923 (0.507-1.680) |
| Vigorous | -0.082 | 0.267 | -0.309 | 0.757 | 0.921 (0.546-1.554) |
| Night sleep duration, |  |  |  |  |  |
| Insufficient |  |  |  |  | 1.000 (Reference) |
| Sufficient | -0.362 | 0.187 | -1.932 | 0.053 | 0.696 (0.482-1.005) |
| Excessive | 0.189 | 0.281 | 0.673 | 0.501 | 1.208 (0.696-2.096) |
| Dash score |  |  |  |  |  |
| ≤20 |  |  |  |  | 1.000 (Reference) |
| 21—24 | -0.055 | 0.204 | -0.271 | 0.787 | 0.946 (0.634-1.412) |
| ≥25 | 0.027 | 0.197 | 0.135 | 0.893 | 1.027 (0.698-1.511) |
| Hypertension |  |  |  |  |  |
| No |  |  |  |  | 1.000 (Reference) |
| Yes | 0.470 | 0.180 | 2.607 | **0.009** | 1.600 (1.124-2.278) |
| Diabetes |  |  |  |  |  |
| No |  |  |  |  | 1.000 (Reference) |
| Yes | -1.106 | 0.380 | -2.914 | **0.004** | 0.331 (0.157-0.696) |
| Overweight/obesity |  |  |  |  |  |
| No |  |  |  |  | 1.000 (Reference) |
| Yes | 0.328 | 0.186 | 1.764 | 0.078 | 1.388 (0.964-1.997) |
| Central obesity |  |  |  |  |  |
| No |  |  |  |  | 1.000 (Reference) |
| Yes | 0.509 | 0.189 | 2.701 | **0.007** | 1.664 (1.150-2.407) |
| OR. Odds Ratio, CI. Confidence Interval; DASH. dietary approaches to stop hypertension; RC. remnant cholesterol; PA. physical activity | | | | | |
