# Supplementary material for: Remnant cholesterol: an independent, dose-dependent risk factor for hyperuricemia in a normolipidemic chinese population
Source: Front Endocrinol (Lausanne). 2026 Jan 12;16:1718817. doi: 10.3389/fendo.2025.1718817 (PMC12832488; doi:10.3389/fendo.2025.1718817)
Supplement: Supplementary file 15 [file Table5.docx]

### Supplementary Table 5

| Variables | β | S.E | Z | *P* | OR (95%CI) |
| --- | --- | --- | --- | --- | --- |
|  |  |  |  |  |  |
| Intercept | -1.250 | 1.226 | -1.020 | 0.308 | 0.287 (0.026 ~ 3.166) |
| RC |  |  |  |  |  |
| 0.14—0.43 |  |  |  |  | 1.000 (Reference) |
| 0.44—0.60 | 0.571 | 0.517 | 1.105 | 0.269 | 1.770 (0.643 ~ 4.876) |
| 0.61—0.89 | 1.179 | 0.506 | 2.329 | **0.020** | 3.252 (1.206 ~ 8.771) |
| ≥0.90 | 0.363 | 0.930 | 0.391 | 0.696 | 1.438 (0.233 ~ 8.898) |
| age/year |  |  |  |  |  |
| 30—44 |  |  |  |  | 1.000 (Reference) |
| 45—59 | -0.120 | 0.461 | -0.260 | 0.795 | 0.887 (0.359 ~ 2.190) |
| 60—79 | -1.161 | 0.821 | -1.414 | 0.157 | 0.313 (0.063 ~ 1.566) |
| Sex |  |  |  |  |  |
| Males |  |  |  |  | 1.000 (Reference) |
| Females | -3.404 | 0.796 | -4.279 | **<.001** | 0.033 (0.007 ~ 0.158) |
| Marriage status |  |  |  |  |  |
| Married/cohabiting |  |  |  |  | 1.000 (Reference) |
| Separated/divorced/widowed/unmarried | -0.537 | 0.812 | -0.662 | 0.508 | 0.584 (0.119 ~ 2.867) |
| Education level |  |  |  |  |  |
| Primary school or below |  |  |  |  | 1.000 (Reference) |
| Junior middle school | -0.122 | 0.636 | -0.191 | 0.848 | 0.886 (0.255 ~ 3.078) |
| High school or above | -0.678 | 0.467 | -1.453 | 0.146 | 0.508 (0.203 ~ 1.267) |
| Occupation |  |  |  |  |  |
| Farmers |  |  |  |  | 1.000 (Reference) |
| Government employees | 1.262 | 0.696 | 1.814 | 0.070 | 3.533 (0.903 ~ 13.822) |
| Workers | 0.753 | 0.645 | 1.167 | 0.243 | 2.124 (0.600 ~ 7.524) |
| Sales staff | 0.229 | 0.677 | 0.338 | 0.735 | 1.257 (0.334 ~ 4.738) |
| Others | 0.235 | 0.724 | 0.325 | 0.745 | 1.265 (0.306 ~ 5.227) |
| Total family income/yuan |  |  |  |  |  |
| ＜20000 |  |  |  |  | 1.000 (Reference) |
| 20,000—59,999 | -0.405 | 0.533 | -0.760 | 0.447 | 0.667 (0.235 ~ 1.895) |
| 60,000—99,999 | -0.200 | 0.608 | -0.329 | 0.742 | 0.819 (0.249 ~ 2.695) |
| ≥100,000 | -0.402 | 0.658 | -0.610 | 0.542 | 0.669 (0.184 ~ 2.432) |
| Smoking status |  |  |  |  |  |
| No |  |  |  |  | 1.000 (Reference) |
| Yes | 0.261 | 0.454 | 0.574 | 0.566 | 1.298 (0.533 ~ 3.162) |
| Dringking status |  |  |  |  |  |
| No |  |  |  |  | 1.000 (Reference) |
| Yes | -0.029 | 0.463 | -0.062 | 0.951 | 0.972 (0.392 ~ 2.407) |
| PA level |  |  |  |  |  |
| Low |  |  |  |  | 1.000 (Reference) |
| Moderate | -1.122 | 1.010 | -1.110 | 0.267 | 0.326 (0.045 ~ 2.360) |
| Vigorous | -0.625 | 0.783 | -0.799 | 0.425 | 0.535 (0.115 ~ 2.483) |
| Night sleep duration, |  |  |  |  |  |
| Insufficient |  |  |  |  | 1.000 (Reference) |
| Sufficient | -0.612 | 0.484 | -1.264 | 0.206 | 0.542 (0.210 ~ 1.401) |
| Excessive | 0.280 | 0.677 | 0.414 | 0.679 | 1.323 (0.351 ~ 4.990) |
| Dash score |  |  |  |  |  |
| ≤20 |  |  |  |  | 1.000 (Reference) |
| 21—24 | -0.710 | 0.590 | -1.203 | 0.229 | 0.492 (0.155 ~ 1.563) |
| ≥25 | 0.307 | 0.468 | 0.656 | 0.512 | 1.360 (0.543 ~ 3.404) |
| OR. Odds Ratio, CI. Confidence Interval; DASH. dietary approaches to stop hypertension; RC. remnant cholesterol; PA. physical activity | | | | | |
